# Monthly Summary

**Published:** 1867-12

**Authors:** 


### MONTHLY SUMMARY.

*A New Process for Preparing Anatomical Specimens.*—Dr. Brunetti, of Padua, who received a gold medal at the Paris Exposition, has generously communicated to the international Medical Congress the following particulars of his valuable invention. The process comprises four several operations, *viz.*, 1, the washing of the piece to be preserved; 2, the *degraissage*, or eating away away of the fatty matter: 3, the tanning; and 4, the desiccation.

1. To wash the piece, M. Brunetti passes a current of pure water through the bloodvessels and the various excretory canals, and then he washes the water out by a current of alcohol.

2. For destroying the fat, he follows the alcohol with ether, which he pushes, of course, through the same bloodvessels, and excretory ducts; this part of the operation lasts some hours.

The ether penetrates the interstices of the flesh, and dissolves all the fat. The piece, at this point of the process, may be preserved any length of time desired, plunging in ether, before proceeding to the final operations.

3. For the tanning process, M. Brunetti dissolves tannin in boiling distilled water, and then after washing the ether out of the vessels with distilled water, he throws this solution in.

4. For the drying process, M. Brunetti places the pieces in a vase with a double bottom, filled with boiling water, and he fills the places of the preceding liquids with warm, dry air. By the aid of a reservoir, in which air is compressed to about two atmospheres, and which communicates by a stop-cock and a system of tubes, first to a vase containing chloride of calcium, then with another heated, then with the vessels and excretory ducts of the anatomical piece in course of preparation, he establishes a gaseous current which expels, in a very little time, all the fluids. The operation is now finished.

The piece remains supple, light, preserves its size, its normal relations, its solid elements, for there are no longer any fluids in it. It may be handled without fear, and will last indefinitely. The discovery is a magnificant one, and the sooner medical schools are provided with full cabinets of natural and pathological pieces the better.—*Medical and Surgical Reporter.*

---

*Preservation of Anatomical Subjects.*—The object which is to be preserved is dipped in a mixture formed by adding to seven parts glycerin, one part brown sugar and half part nitre, until a slight deposit begins to be perceived on the bottom of the vessel. Putrefaction is thus entirely prevented, the object when taken from the solution being perfectly rigid, but by hanging it in a warm and dry place, the muscles and articulations will recover all their pliancy.

---

*Cryptopia.*—A new alkaloid in opium has been discovered by Messrs. T. & H. Smith, which they have named cryptopia. The formula of this new alkaloid is $C_{23}\ H_{25}\ NO_5$. Its primary form is a hexagonal prism, and it is obtained in this condition if crystallized slowly in a tube from its alcoholic solution. Messrs Smith have succeeded in making the sulphate, muriate, nitrate, the subacetate and the acetate; these all crystallize in distinct forms, but the alkaloid itself has much better crystalline forms than any of its compounds. Four or five tons of opium only yield five ounces of muriate of cryptopia.

---

*Malarial Neuralgia Cured by Hypodermic Injections of Quinia.*—The *New York Medical Journal* contains the records of six cases of this disease treated in the New York Hospital. The solution employed by Dr. Seguin was prepared as follows: Take of sulphate of quinia, 60 grains; dilute sulphuric acid, 40 minims; distilled water, one fluid ounce. Mix; make a solution, and filter with the greatest care. Thirty-five minims are equal to 4 grains of quinia. The immediate effect of injecting this solution was a severe, burning pain, due to the acid; it passed off, usually, within twenty minutes. Pain and hyperesthesia were arrested, and in some instances slight though distinct local anesthesia produced. No abscess or other unpleasant consequence followed any of the seventy-eight injections used in the six cases. Five of the cases had histories of intermittent fever; in four instances the pain was in the region of the spleen or epigastrium: in one it was in the back, running up to the right shoulder, and the last suffered from sciatica of the left thigh. The amount of the solution injected varied from xviii to xxxv minims. Quinia by the mouth, together with a mixture of bark and iron, were daily administered.

---

*Heat as a Resuscitating Agent.*—Dr. J. Richardson, in the *Am. Jour.*, urges from his observation and experiments the importance of direct Heat as an agent in the restoration of still-born infants, in cases of asphyxia from drowning, hanging, or the inhalation of noxious gases, especially the vapor of chloroform. He advises not merely *warming* the surface but artificially warming the blood within the limbs of persons apparently dead, and then propelling by frictions towards the heart as rapidly as possible. The heart's pulsations in many instances really take place, feebly, for a considerable time after being undiscoverable by external observation. The heat should approximate *roasting* as nearly as may be without positive destruction of the tissues. Mere vesication he thinks ought not to be considered more than a minor evil in comparison with the cessation of life.

---

*Extraordinary Obesity.*—A woman named Hogan, wife of a comfortable farmer living at Kilmastulla, Ireland, died recently from obesity. Mrs. Hogan in her youth, showed symptoms of attaining more than ordinary proportions, and she continued to increase in size until, at the time of her death, she had reached the extraordinary weight of forty-eight stone (672 pounds) ! She was, for the last few years of her life, scarcely able to walk, and or some time past entirely confined to her bed.

---

*Death from Swallowing Two Ounces of Chloroform.*—Dr. D W. Stormont, of Topeka, Kansas, reports (*Leavenworth Medical Herald*) a case of suicide by the internal administration of chloroform. The patient was 26 years of age, and in good health at the time. He swallowed two ounces of undiluted chloroform, at a single draught. In three minutes after he had laid himself composedly down, he could with difficulty be aroused from the stupor into which he was rapidly sinking; and though he could not speak, he indicated that he had severe pain in the region of the stomach. In five minutes, he was entirely unconscious and breathing stertorously. He died, in just one hour after taking the draught. Medical assistance, from some cause, did not arrive until a few minutes before he died, and nothing was done to counteract the effects of the poison. At the *post mortem* examination, the surface generally was livid; the face, neck, chest, and nails very much so. Bloody froth was issuing from the mouth and nostrils. On opening the chest, both lungs were found to be dark externally, and fully distended. They were uniformly congested with dark, liquid blood, and the posterior portions were perfectly engorged with it. Both sides of the heart were nearly full of black, uncoagulated blood ; the liver and spleen both normal externally, but somewhat softened, and filled with dark, liquid blood. The œsophagus was congested. The stomach, at the cardiac end, and along the greater curvature, and half way up each side, was discolored externally, dotted over with ecchymosed-looking patches, giving it a mottled appearance. It contained two or three ounces of a lightcolored liquid, which had a slight odor of chloroform. At the cardiac end, internally, and along the bottom, nearly to the pyloric end, the mucous membrane was of a dark-red color, softened, and easily peeled off with the thumb-nail. Up the sides, it was of a brighter red, speckled appearance, and not softened. The intestines were healthy. Circumstances prevented the extension of the examination, which is much to be regretted. The reporter of the case closes with the following remarks: —"Recoveries are recorded from drinking two ounces, or even more, of chloroform, but active measures were used—as the stomach-pump, emetics, stimulants, internal and external artificial respiration, galvanism, etc. As an internal stimulant, the spirits of ammonia, or the carbonate of ammonia, is the best. Very dangerous symptoms have been produced by half an ounce, and death has been caused by one ounce, Dr. Stille says:—'When death has been produced by the internal use of chloroform, its local irritant action has evidently been the chief cause of the fatal result.' In this case, death followed too soon to have been produced in this way. It was more probably caused by the action of the poison on the blood and the cerebro-spinal system, as in prolonged inhalation of the vapor. A peculiarity of this case, is the shortness of the time between taking the chloroform and death, as compared with other fatal cases reported."—*Chicago Medical Examiner.*

---

*New Anaesthetics.*—Dr. A. E. Sansom, at the close of an article upon the action of the tetrachloride of carbon, which he introduced to the profession as an anæsthetic, sums up the result of his experience thus:—"From what I have now said, it will be gathered that I do not consider the tetrachloride to be the *summum bonum* of an anæsthetic. So far as its earlier stages are concerned, it is all we want: it is stimulant, anodyne, hypnotic, and it produces no adverse sign. But for the anæsthesia necessary for the performance of surgical operations, as well as for any prolonged employment, I consider it altogether undesirable. The accidents of its physical condition, its ponderous vapor, its insufficient volatility for the system readily to disembarrass itself of it, are so many reasons for its non-employment in anything like large doses." Of his experiments in correcting its bad effects by mixture with chloroform, in various proportions, he says:—The conclusion to which I have come from my experiments has been that, the greater the proportion of the tetrachloride, the greater the tendency to spasmodic, jerking inspiration, and the greater the tendency to a deepening of the narcosis after removal from the atmosphere. A small proportion of the tetrachloride seems to me to lend safety to the action of chloroform. On this point we want further experiments; but I think we shall find a mixture of one part of tetrachloride of carbon in six of chloroform a safe, as it certainly is an agreeable anæsthetic. Apropos of anæsthetics, Dr. Richardson announces the bi-chloride of methylene, $C_2 H_2 Cl_2$, to be an anæsthetic far superior to chloroform, in that it equals or surpasses its advantages without its dangers.—*Medical Gazette.*

---

*Aluminium—Its Properties and Uses.*—The discovery of this metal dates back only to 1827, when Wohler, a German chemist succeeded in extracting it from clay. It is a white metal, not like silver, but having a bluish tinge. Its specific gravity is from 2.5 to 2.67 according to its purity. It is considerably lighter than flint glass, being, as seen above, only about two-and-a-half times heavier than water. Bulk for bulk it is four times as light as silver and a little more than quarter the weight of copper. It is nearly as hard as iron, but can be softened by annealing; has great rigidity and tenacity; can be turned, chased, and filed with ease, never clogging the file; and can be drawn into wire as fine as a hair and rolled or beaten into sheets whose thinness can be surpassed only by those from gold or silver.

For mustard and egg spoons it would be an excellent material, as, unlike silver, it is not affected by sulphureted hydrogen or other sulphureted compounds. It retains its lustre in the ordinary atmosphere and is not affected by boiling water, diluted sulphuric, or strong nitric acid, which attacks silver, but has no action upon aluminium when cold, and it is not affected when plunged into melted niter, potass, or sulphuret of potassium, a test which even gold or platinum cannot withstand. It is dissolved, however, in muriatic acid and has a powerful attraction for chlorine.

It has been used in France and England for ornamental purposes, as finger rings, brooches, chains, etc. A cup made of it, although very thin, was not indented by falling from the hand to the pavement, These peculiar properties would seem to make it a proper material for light field guns, cuirasses, helmets and coins, but for the cost of extracting it from its earthy base of argil or clay.

When the inventive genius of man has discovered a cheap and rapid process of extracting aluminium we may expect it to assume a much more important position in the useful, as well as the ornamental arts, than it occupies at present. A beautiful compound is now manufactured in France and England composed of aluminium 10 and copper 90 parts. We have seen a paper cutter, the blade and handle made of this, which had a beautiful yellow or deep straw color, was elastic, tough, and of a very fine finish. Its color is more grateful to the eye than gold and its lustre brilliant. The earth metals, of which aluminium may be considered the head, will in time become as valuable for use as they are now for ornament or for the purpose of the chemist.

---

